# Multi-Fault Detection and Classification of Wind Turbines Using Stacking Classifier

**DOI:** 10.3390/s22186955

**Published:** 2022-09-14

**Authors:** Prince Waqas Khan, Yung-Cheol Byun

**Affiliations:** Department of Computer Engineering, Jeju National University, Jeju-si 63243, Korea

**Keywords:** wind turbines, fault detection, stacking ensemble classifier, AdaBoost, K-nearest neighbors, logistic regression

## Abstract

Wind turbines are widely used worldwide to generate clean, renewable energy. The biggest issue with a wind turbine is reducing failures and downtime, which lowers costs associated with operations and maintenance. Wind turbines’ consistency and timely maintenance can enhance their performance and dependability. Still, the traditional routine configuration makes detecting faults of wind turbines difficult. Supervisory control and data acquisition (SCADA) produces reliable and affordable quality data for the health condition of wind turbine operations. For wind power to be sufficiently reliable, it is crucial to retrieve useful information from SCADA successfully. This article proposes a new AdaBoost, K-nearest neighbors, and logistic regression-based stacking ensemble (AKL-SE) classifier to classify the faults of the wind turbine condition monitoring system. A stacking ensemble classifier integrates different classification models to enhance the model’s accuracy. We have used three classifiers, AdaBoost, K-nearest neighbors, and logistic regression, as base models to make output. The output of these three classifiers is used as input in the logistic regression classifier’s meta-model. To improve the data validity, SCADA data are first preprocessed by cleaning and removing any abnormal data. Next, the Pearson correlation coefficient was used to choose the input variables. The Stacking Ensemble classifier was trained using these parameters. The analysis demonstrates that the suggested method successfully identifies faults in wind turbines when applied to local 3 MW wind turbines. The proposed approach shows the potential for effective wind energy use, which could encourage the use of clean energy.

## 1. Introduction

The global energy system is recognized as a significant contributor to greenhouse gas emissions. Sustainable development of low carbon emissions is required for decarbonization. The need for modern energy has taken an essential step toward renewable energy. Due to its positive benefits, wind power is attracting a significant amount of interest from investors in renewable energy sources. Wind energy is a clean, pollution-free renewable energy source with excellent growth potential [1]. Wind’s kinetic energy does not produce carbon dioxide and provides clean, reliable electricity. Over the last few years, the installed capacity of wind power has grown exponentially. However, most wind farms are located in remote areas with challenging environments such as mountains, deserts, or oceans. Many components of wind turbines, such as fans, bearings, gears, and generators, are at risk of failure, leading to higher maintenance and operating costs for wind turbines. Continuous health monitoring and maintenance can improve the reliability of wind turbines and prevent catastrophic accidents. Therefore, monitoring the condition of wind turbines and identifying faults is necessary and valuable for maintenance and planning, which can reduce economic losses and promote the development of the wind power industry.

Due to high-quality complex structures, high maintenance costs, and an increase in wind turbine installations worldwide, wind turbine operation and maintenance (O&M) technologies are now required [2]. In addition, offshore wind power sources, especially floating wind turbines, are rapidly evolving, significantly increasing the hassle and cost of O&M. Operating and maintenance costs make up a large portion of the total annual cost of wind turbines. For a new turbine, the cost of O&M can easily be as high as 20–25 percent of the total cost of an aircraft producing kWh during the turbine’s life. If the turbine is relatively new, the component may be only 10–15 percent, but it may increase by at least 20–35 percent during the turbine’s life. As a result, more attention is paid to O&M as manufacturers try to significantly reduce costs by designing new turbines that require less service testing and shorter turbine downtime. The consistency and timely maintenance of wind turbines can enhance their reliability and performance [3]. Much research has focused on monitoring control and early detection of faults through Supervisory Control and Data Acquisition (SCADA). Detecting the condition or malfunction of wind turbines is an inexpensive solution to reduce maintenance costs and revenue loss due to component malfunction. The design of debugging deployment turbines has a more significant impact on life expectancy. Older turbine gearboxes did not take advantage of advances in engineering and were still at risk of decay and wear and tear. Several artificial intelligence solutions have been proposed that allow technicians to predict and identify turbine faults, assist in diagnosis, and determine when safety precautions should be taken. Machine learning solutions have become popular in many fields, and their use in wind turbines has yielded excellent results.

This paper proposes a new AdaBoost, K-nearest neighbors, and logistic regression-based stacking ensemble (AKL-SE) classifier to classify the faults of the wind turbine condition monitoring system. The work is based on the pre-identified status of the fault obtained from the condition monitoring system (CMS). Using the proposed system, the need and expenses of CMS can be cut off. A stacking ensemble classifier combines different classification models to enhance the model’s accuracy. We have used three classifiers, AdaBoost, K-nearest neighbors, and logistic regression, as base models to make output. The output of these three classifiers is used as input in the meta-model [4]. To improve the data validity, SCADA data are first preprocessed by cleaning and removing any abnormal data. Then, using the Pearson correlation coefficient, the input variables are chosen. The stacking ensemble classifier was then trained using these parameters. The analysis demonstrates the effectiveness of the proposed method in identifying wind turbine anomalies after being applied to several 3 MW wind turbines. In the eleven months of data, there were more than forty different types of faults, but only a small fraction occurred frequently enough for a classifier to be trained to identify them. We classify five different types of faults: feeding faults, excitation errors, generator heating faults, malfunctioning air cooling, and mains failure. The proposed AKL-SE outperforms the state-of-the-art methods for intelligent multi-fault classification in the wind turbine.

### 1.1. Contributions

The significant contributions of this article can be summarized as follow

- An AdaBoost, K-nearest neighbors, and logistic regression-based stacking ensemble (AKL-SE) classifier is introduced.
- We integrated SCADA data with status data for fault detection and employed data and predictive analytics techniques for data obtained from wind turbines.
- We performed a comparison with state-of-the-art ML models and different combinations of ensemble models.

### 1.2. Article Organization

The remainder of the article is arranged as follows. Section 2 presents the review and analyses of related publications. Section 3 presents the proposed methodology and explanation of the models used. Section 4 explains the exploratory data analysis. Section 5 provides the results and also covers the comparison with the latest machine learning models. Finally, we conclude this article in the last conclusion section.

## 2. Literature Review

Wind power is an essential renewable energy source due to technological advances and cheapness. Wind turbines (WT) are essential for wind power generation systems [5,6]. Defects in various parts of wind turbines should be eliminated for better performance. Vibration testing is an integral part of monitoring the condition of wind turbine components [7]. In the article by Bodla et al. [8], a test was performed using average and incorrect WT vibration data to monitor low position. The purpose is to monitor the condition of the wind turbine for immediate fault predictions so that the turbine can be configured for immediate better performance and longer service life. Quality monitoring and evaluation are essential for proper functioning wind power generation systems. In the study by Malik et al. [9], health conditions were assessed for three different fault types using K-nearest neighbors (KNN) algorithms. To decompose the raw signal, they used a separate estimation of the wave function called the Meyer wavelet function. The wind generator used 21 full-featured templates to classify imbalance errors. They compared the proposed method to a multilayer perceptron (MLP). The proposed approach results and various comparisons can serve as an essential tool for WTG error detection.

The approaches used in the study by Lima et al. [10] enable information mining from easily accessible SCADA data in order to identify potential abnormalities when wind turbines run. With this system monitoring the turbine parts, it is feasible to see problems before they arise and take appropriate action, minimizing downtime and maintenance expenses. A customized deep learning model was suggested by Chatterjee et al. [11] for anomaly prediction and transparent decision-making. It was extended to transfer learning from one domain to another, eliminating the need for training in the new domain.

The converter can quickly fail as a vital component of the transmission system. In order to isolate vulnerable sums during the integration process, the best model parameters for each SVM can be found by repeating BSA optimization in the article by Zheng et al. [12]. To improve fault detection accuracy for wind turbines, a method of correction of fault correlation with wave change and compression detection theory has been proposed. It also uses the AdaBoost-SVM wind turbine converter for diagnostics. Based on the compression observation theory, the estimated coefficient of the wave is quantitatively reduced to obtain the measured signal. The target of the orthogonal adjustment is the algorithm tracking vector and then the error multiplier vector. AdaBoost-SVM’s advanced classification for error detection has introduced error detection vectors.

The study by Wu et al. [13] presented a wind turbine diagnostic method based on the XGBoost and ReliefF algorithms that used SCADA data to improve the accuracy of wind turbine diagnostics. This article provides solutions to wind turbine malfunction. The advantage of high accuracy in the algorithm was verified by comparison with the state-of-the-art models.

Wind energy is converted into electrical energy by a rotating blade attached to a generator [14]. Due to environmental conditions and extensive construction, the blades suffer from many defects and lack of productivity [15]. The time of inactivity can be reduced if their condition is checked periodically using monitoring techniques. Feature extraction, feature selection, and error classification have been considered machine learning issues. The study by Joshua et al. [16] provided an explanation based on the algorithm of vibration signals for analyzing wind turbine aircraft conditions. A model was created using the data formatting technique from the vibration data obtained. A logistic model tree (LMT) algorithm was used to learn and classify different aircraft locations. Samples were tested ten times under cross-verification, and 90.33 percent accurate classification samples were found. The error rate was relatively low and can be considered blade debugging. Therefore, the tree logistics model is available mainly for monitoring wind turbine blades to reduce idle time and provide continuous wind power. Renewable energy sources such as wind power are plentiful. The reliability of wind turbines is essential to maximize wind power. Vibration signals on the rotating side of wind turbines were average, not Gaussian, and instability and malfunction patterns were generally minimal. With these issues in mind, The article by Wenyi et al. [17] designed and proposed a method for detecting wind turbine faults based on the grouping of the diagonal spectrum and binary tree support-vector machine (SVM). First, the input properties of the diagonal spectrum vibration machine were considered vectors. Second, a self-regulating neural network mapping feature was introduced to create error feature group sampling and a cluster binary tree. Numerous error classifications were made for practice and sample testing. The diagnostic analysis results confirm that this paper method is effective and efficient. Impact classification effects were RBF neural network methods, and higher accuracy could be achieved in better and fewer sample cases than for traditional SVM methods.

The study by Jiang et al. [18] focused on a survey of the proposed architecture and the multifaceted properties of low-turn vibration signals for troubleshooting WT gearboxes under different operating conditions. Unlike traditional methods, when the emission and classification of features are individually designed, the design’s purpose was to automatically determine the characteristics of the desired error of the vibration signal. The proposed multiscale convolutional neural network system was evaluated by testing the test machine on the WT gearbox. Experimental results and extensive comparative analysis of traditional CNN and traditional multidimensional extractors demonstrated the effectiveness of the proposed method. The accuracy could be enhanced by increasing the features [19]. They verified the scale of their proposed MSCNN with an accurate mass WT gearbox. In addition, they further explored analytical methods based on unbalanced multidimensional presentations to significantly reduce the impact of the distribution of diagonal data between standard and inaccurate data, leading to learning algorithms for error diagnosis to significantly improve performance.

Modern debugging and rating systems have become necessary to achieve wind turbines’ desired reliability and efficiency [20]. In the work by Vidal et al. [21], sampling frequency was increased, and database multi-error detection monitoring and current sensor classification (in all commercial wind turbines) monitoring controls and data acquisition (SCADA) systems were monitored without the use of special equipment. A high-quality wind turbine was used. They understood that there were several defects in wind turbine actuators and sensors. First, the SCADA measurement groups were preset by a feature change based on analyzing multiple key components and sample base openings. Then, classification based on 10-fold cross-verification SVM was applied. The result was a uniquely trained classification that can solve all the errors analyzed by calculating only a set of features from the data for evaluation. As a result, their proposed approach is better than the other methods.

Time series data on the monitoring and debugging of wind turbines and other power systems are widely used [22], where long-term reliability is essential for developing classification features. Lei et al. [23] identified errors in time series signals based on long shorts. They introduced a new method using the long short-term memory (LSTM) model to learn features directly from multiple variable time series data and gain long-term possibilities through recurring behaviors and LSTM gate mechanisms. Errors can be efficiently classified by one or more sensors using raw time-series signals, and the performance of modern technologies is more efficient. Further, the sustainability of the proposed structure can be verified by testing it on smaller datasets with limited data. This could be enhanced by using a CNN as part of preparations to acquire local features to enhance the functionality of the proposed structure.

Classification of multi-fault detection is a challenging task due to weak faults [24], especially in wind turbine gearboxes with different gears and bearings [25]. The study by Teng et al. [26] analyzed the vibration signals coming from a real multi-fault wind turbine gearbox with catastrophic failure. The complex waveform can provide a multiscale enveloping spectrogram for simultaneous decomposition and distortion of signals. Using this method, testing multiscale enveloping spectrogram disks on different scales can quickly determine the faulty characteristics of the mounted holder under compression force. Unplanned or unresponsive maintenance of wind turbines due to component failure can lose a significant amount of time and revenue [27]. For this purpose, it is necessary to maintain it before it is needed. By constantly monitoring the health of the turbine, it is possible to eliminate the need for periodic inspections, identify underlying defects, and adjust maintenance schedules as needed. Efforts have been made to develop a condition monitoring system (CMS) based on detecting expensive vibration [28,29] or oil analysis sensors [30,31] in turbines. Instead, critical analyses of existing data from the turbine’s SCADA system can provide essential insights into turbine performance at a low cost. The study by Leahy et al. [32] examines a new method for classifying and predicting turbine faults based on SCADA data. The data were taken from a SCADA system in southeastern Ireland. Error classification works on three levels: distinguishing between error/error-free operations and classifying a specific error. Error and warning data were analyzed using simple and forced curves to identify the duration of filtering and error activation. The results were good and showed that perfect memory and accuracy could differentiate between error and error-free operations, but the F1 score dropped due to poor accuracy.

## 3. Methodology

**Figure 1:**
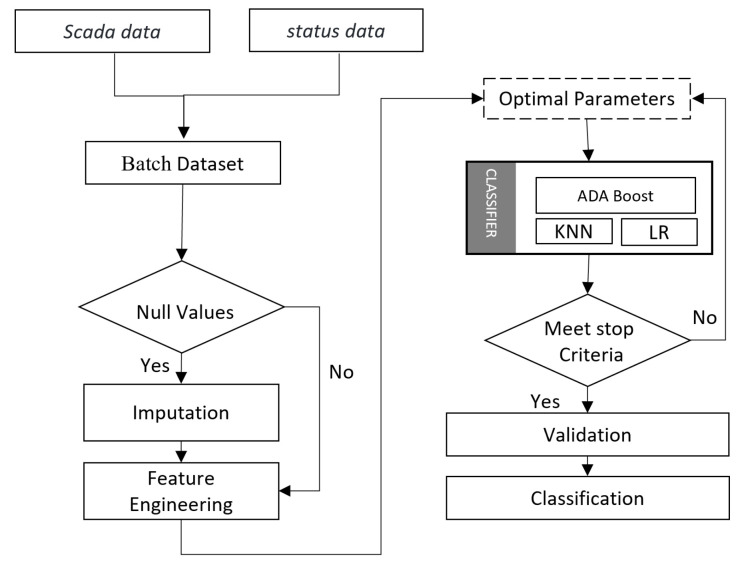
Flow diagram.

Classification of multi-fault detection is a challenging task due to weak faults [24]. Different machine learning algorithms have their positive and negative sides [33,34]. We have proposed a stacking ensemble classifier that utilizes different classification models to enhance model accuracy. We have proposed a new AdaBoost, K-nearest neighbors, and logistic regression-based stacking ensemble (AKL-SE) classifier. Figure 1 shows the flow diagram of the proposed methodology. We combine SCADA data with the status data obtained from the wind energy converter (WEC). WEC status data corresponds to a status message directly related to the turbine itself [35]. We preprocessed the batch data and applied feature engineering techniques. Then we used a set of parameters to train the stacking ensemble classifier. Finally, we used different evaluation metrics for the validation to classify the fault.

### 3.1. Stacking Ensemble Classification

**Figure 2:**
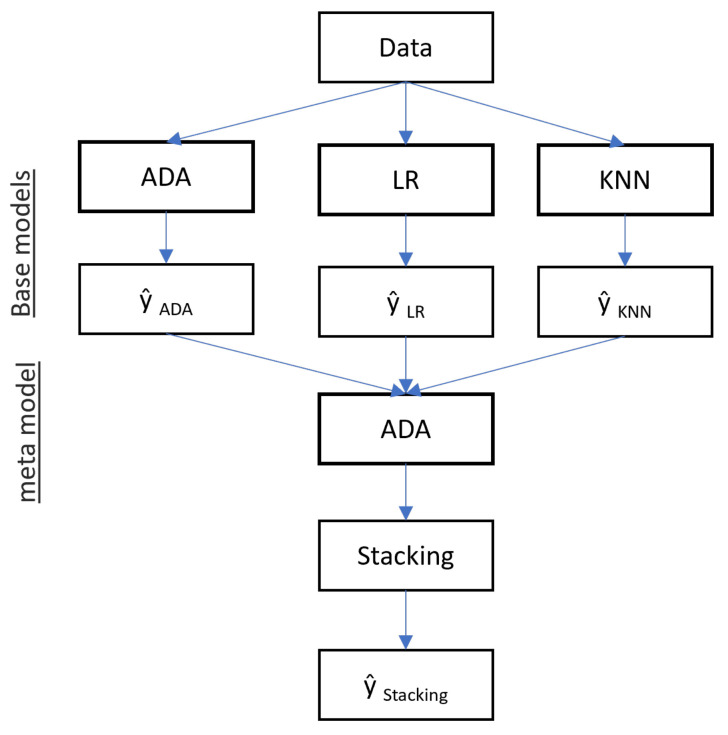
Stacking ensemble classifier model.

Stacking ensemble classifier methods is a method for different classification models to enhance the model’s accuracy [36]. Stacking is one of the ensemble methods. We have used three classifiers for the meta-models at level 0 to make output. The output of these three classifiers is used as input in level 1 of the meta-model classifier. Figure 2 shows the base and meta-models of the proposed AdaBoost, K-nearest neighbors, and logistic regression-based stacking ensemble (AKL-SE) classifier model.

We used the “pycaret” Python library to obtain the training accuracy of different models. Then we selected the top three models and used them for stacking. Table 1 summarizes the hyperparameter settings for the base models: AdaBoost, K-nearest neighbors, and logistic regression. The AdaBoost classifier is used as a meta-model.

### 3.2. AdaBoost

AdaBoost is short for Adaptive Boosting. It is a statistical classification meta-algorithm [37]. AdaBoost works by systematically applying a weak classification to the training dataset that has been reconsidered by re-weighting [38]. It can be used with many other types of learning algorithms to improve performance. Algorithm 1 explains the pseudocode for AdaBoost training, where we assign every observation $x_{i}$ an initial weight value and *n* represents the total number of observations. The first weak decision model is trained, which is usually a decision tree model for every observation. If the prediction is correct, $w_{i}$ is increased; otherwise, it is decreased. Then, we train another weak model where greater-weight observations are given more priority.

**Algorithm 1** Pseudo code for AdaBoost training**Require:** Initialize weight to $x_{i}$ **Require:** Initialize $W_{i}=1/n$ Train first weak decision tree model **for** Each observation $x_{i}$
**do**  **if** pred ≠ correct **then**   $W_{i}++$  **else**   $W_{i}--$  **end if** **end for** Train second weak model with greater weights **return** pred

### 3.3. Logistic Regression

Logistic regression is a classification algorithm that is used to assign observations to a separate class group. In contrast to linear regression, which produces continuous numeric values, logistic regression translates its output into the use of the logistic sigmoid function. A probability value can be mapped to two or more separate classes. The goal is to choose parameters that maximize likelihood (Equation (1)) and the partial derivative of the log-likelihood (Equation (2)) corresponding to each parameter [39].

(1) $$p\left(y_{i}=1|{x_{i}}^{T}w\right)=g\left({x_{i}}^{T}w\right)=1/\left(1+exp\left(-{x_{i}}^{T}w\right)\right)$$

(2) $$L\left(w\right)\;=\sum_{i=1}^{n}\;y_{i}\log\left(g\left({x_{i}}^{T}w\right)\right)+\left(1-y_{i}\right)\log\left(1-g\left({x_{i}}^{T}w\right)\right).$$

where $p(y_{i})$ represents the likelihood function and $L\left(w\right)$ represents the log-likelihood function [40]. The training record is denoted by a matrix $X={\left(x_{1},x_{2},...x_{n}\right)}^{T}$, and $X={\left(x_{i}^{1},x_{i}^{2},...x_{i}^{m}\right)}^{T}$ shows each row vector. Corresponding labels to each record are represented by *Y*, where $Y={\left(y_{1},y_{2},...y_{n}\right)}^{T}$. The parameters $W={\left(w_{1},w_{2},...w_{n}\right)}^{T}$ can be optimized by training the model through $L\left(w\right)$.

### 3.4. K-Nearest Neighbor

K-nearest neighbors (KNN) is a supervised analytics algorithm used for classification and regression problems. The rule is to find the number of pre-determined training samples close to the new point and predict the training samples’ labels [41]. Algorithm 2 represents the pseudocode for K-nearest neighbors training. It starts with the initializing of $k,func,target,data_{t}$, where *k* represents the user-defined constant, the number of closest training data, $data_{t}$ represents all training data points, target represents a new point, and func represents functions used to get the target label. The first step is calculating the Euclidean distance between the new point and all training data. The next step is to pick the top-K closest training data. The most common label of these labels, as a result, is picked and returned.

**Figure 3:**
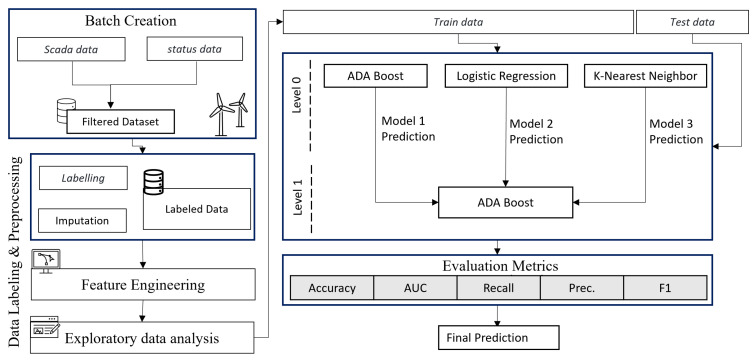
Structure of the proposed methodology.

Figure 3 represents the structure of the proposed methodology. A 3 MW direct-drive turbine in the South of Ireland that provides power to a sizable manufacturing facility close to the coast provided us with benchmark SCADA data. Data come from the turbine SCADA system in the form of 10-minute operational and instantaneous alarm system data.

**Algorithm 2** Pseudo code for K-nearest neighbors training**Require:** Initialize $k,func,target,data_{t}$ **Require:** Initialize neighbors=[] Train first weak decision tree model **for** Each observation $data_{t}$
**do**  distance = euclidean distance (data[:−1],target)  calculate euclidean distance  append neighbors **end for** pick the top-K closest training data take the most common label of these labels **return** labels

In order to properly train a classifier, the data must be correctly labeled [32]. In this paper, we classified samples as specific fault or fault-free. We labeled the data according to the faults, where status number 62 represents feeding fault or load shedding, fault 80 represents excitation error due to an overvoltage DC-link, fault 228 is for a timeout warning message or malfunction in air cooling, Fault 60 is for mains failure and start delay, and fault nine is for generator heating due to the hygrostat inverter. After performing feature engineering and exploratory data analysis, the data were divided into two parts. One was for training the staking ensemble classifier, and the other was for testing. We used different evaluation metrics, including accuracy, area under the curve (AUC), recall, orecision, and F1 score, to evaluate the accuracy of the proposed model.

## 4. Data Analysis

This section covers the exploratory data analysis of SCADA data. The data used in this study were gathered from a 3 MW direct-drive turbine that powers a sizable manufacturing facility close to the coast in the south of Ireland [42]. Data come from the turbine SCADA system in the form of 10-min operational and instantaneous alarm system data. The data cover 11 months from May 2014 to April 2015.

**Figure 4:**
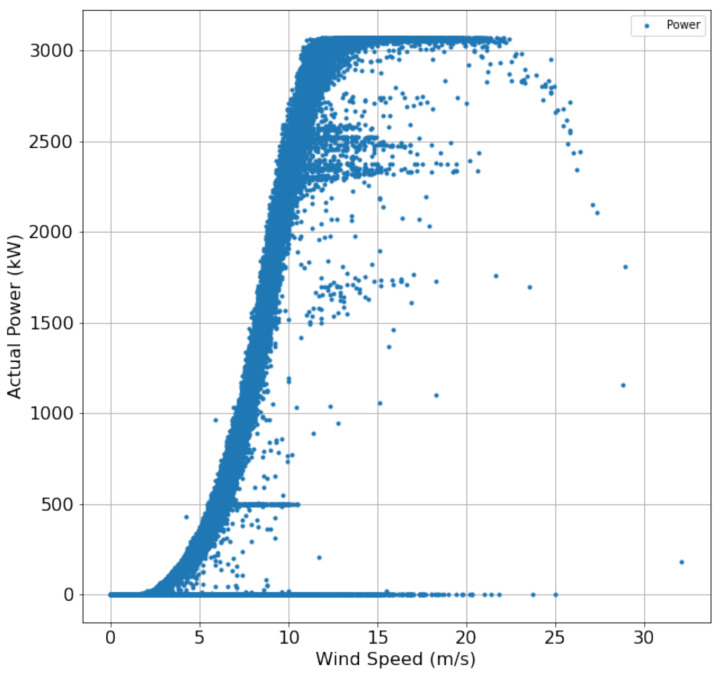
Relation ship between power and wind speed.

Figure 4 shows the relationship between power and wind speed, where *x*-axis represents the wind speed in m/s, and the *y*-axis represents the active power in kwh. An increment in power is observed with the increase in wind speed. However, there are some outliers where the power is zero, even with a high wind speed.

Two distinct datasets, WEC Status data and RTU Status data, save the number of normal and abnormal operation states. The WEC (wind energy converter) status information relates to a message that is specific to the turbine. At the point of connection to the grid, RTU data corresponds to power control data, such as active and reactive power. There are “main status” and “sub-status” codes assigned to each status. Any main WEC status code above 0 denotes erroneous behavior but does not necessarily indicate a fault; for example, status code 2 denotes “lack of wind”. Only active and reactive power set-points are addressed by statuses in RTU; for example, status 100:82 corresponds to limiting the active power output to 82 percent of its actual current output.

**Figure 5:**
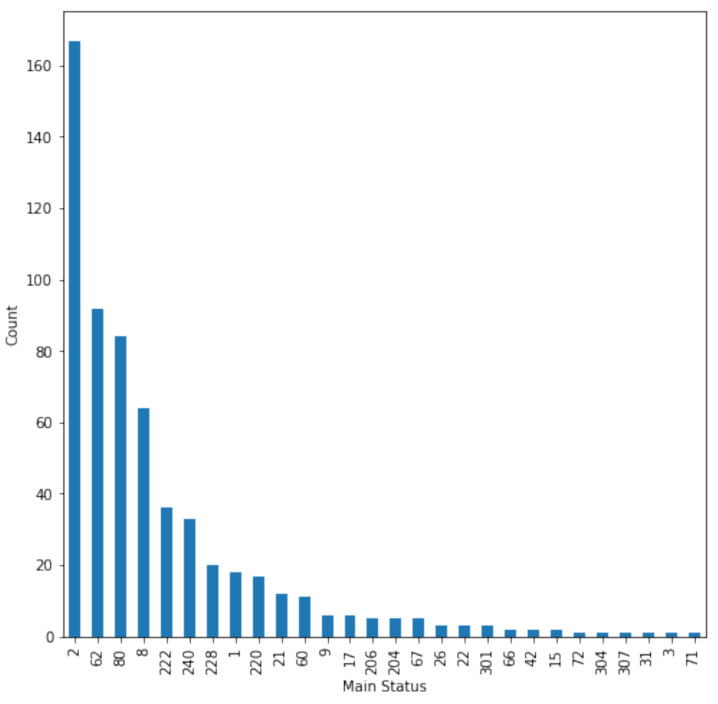
Count of all status readings.

Figure 5 represents the count of all statuses from the wind energy converter in SCADA data. Any main status code of zero indicates that the turbine is generating or spinning up to generate, while anything above zero represents either faulty or reduced operation. The turbine is available to operate but not producing power due to weather, grid, or other events, e.g., status code 2—“lack of wind”.

**Figure 6:**
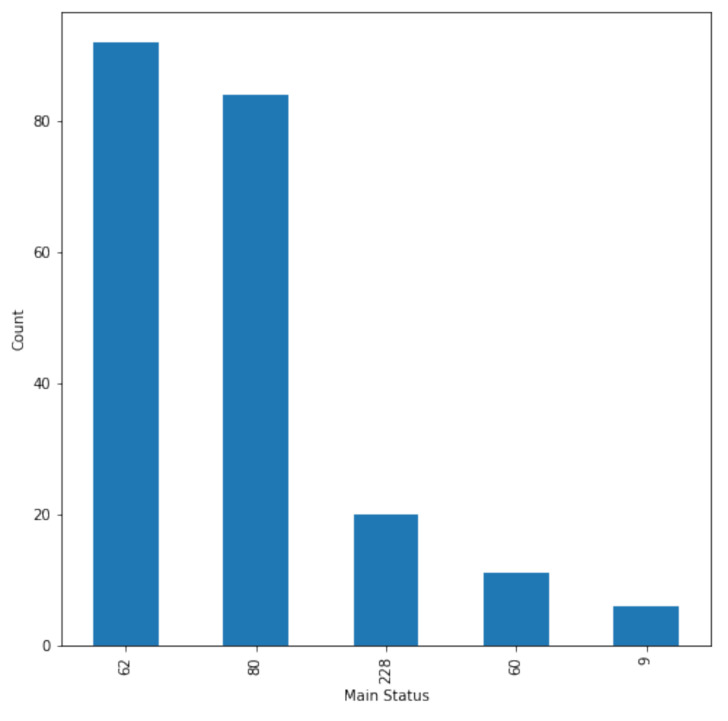
Count of faulty status.

Any “main status” above zero denotes unusual or possibly unusual behavior, but it is not always a fault. In the eleven months of data, there were more than forty different types of faults, but only a small fraction was common enough for a classifier to be trained to identify them. Figure 6 shows the count of faulty status readings. Status number 62 represents a feeding fault or load shedding. Fault 80 represents excitation error due to an overvoltage DC-link. Fault 228 is for a timeout warning message or a malfunction in air cooling. Fault 60 is for mains failure and start delay, and fault nine is for generator heating due to the hygrostat inverter.

**Figure 7:**
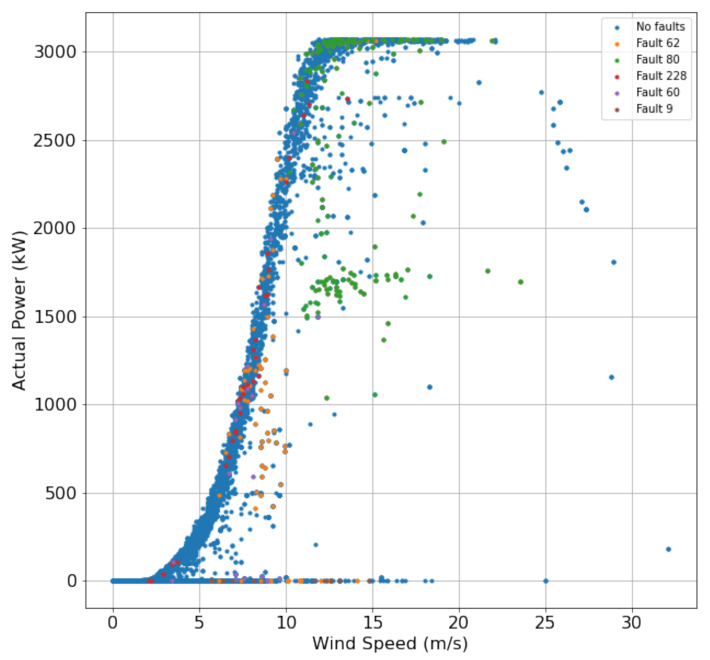
Power and wind speed with respect to non-faulty status and all individual faults.

Figure 7 shows the relationship between power and wind speed concerning non-faulty and all individual fault operations. Blue dots represent no faults, orange dots represent fault 62, green dots represent fault 80, red dots represent fault 228, purple dots represent fault 60, and brown dots represent fault 9.

**Figure 8:**
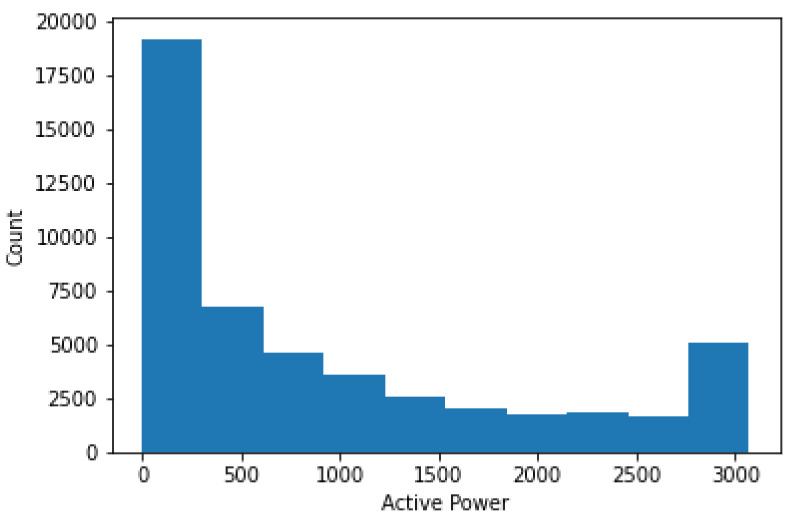
Count of power according to active power range.

Figure 8 shows the count of power. The power in the range of 0–300 has the highest count, whereas 1900–2100 has the lowest count.

**Figure 9:**
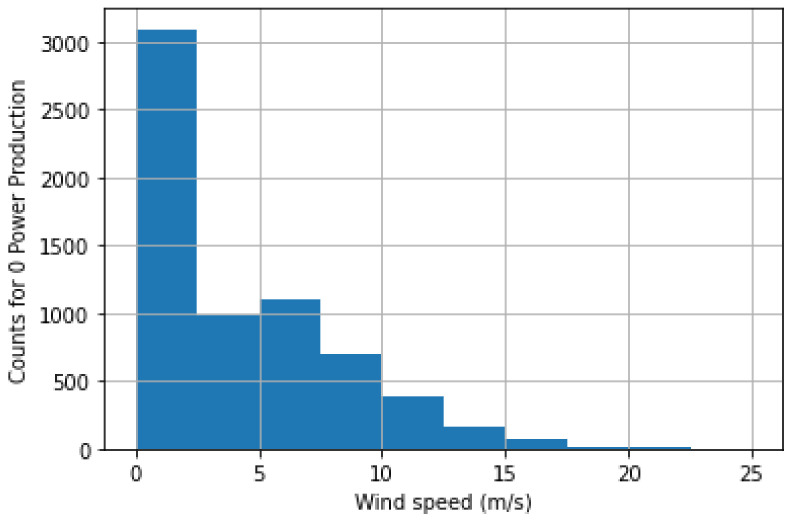
Count of records where wind speed was recorded, but power was zero.

Figure 9 shows the count of records where wind speed was recorded but the power was zero. The count is at its maximum in the wind speed range of 0–2.5 m/s and at its minimum in the range of 22.5–25 m/s.

**Figure 10:**
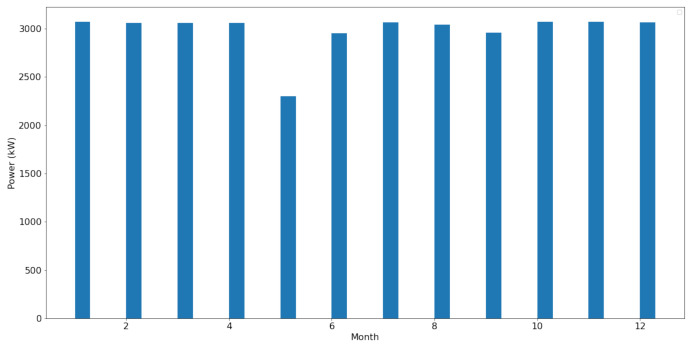
Power recorded according to each month.

Figure 10 shows the power recorded according to each month. May has the lowest power recorded.

## 5. Results

This section consists of different evaluation metrics. Different tabular and graphical ways are used to evaluate the performance of the proposed model. We have used a correlation diagram (Appendix A), confusion matrix, and receiver operating characteristics curve for a graphical representation of performance. Accuracy, precision, recall, and F1 score are used to compare with other state-of-the-art classifiers.

### 5.1. Confusion Matrix

**Figure 11:**
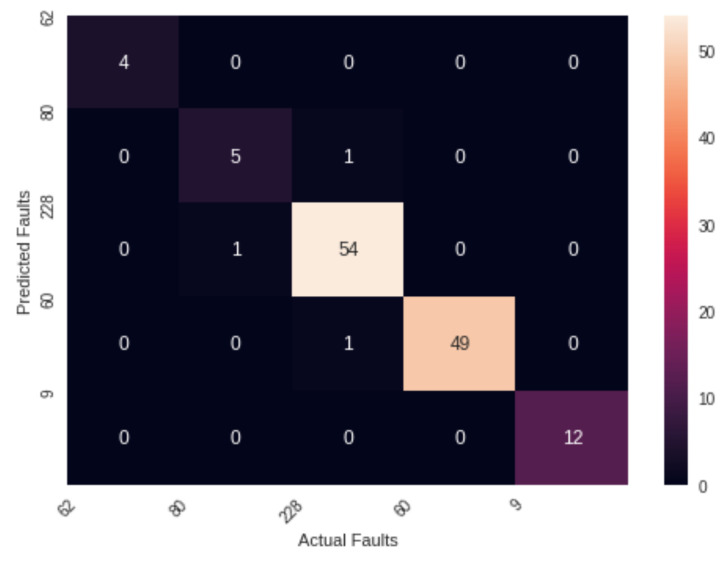
Confusion matrix of faults.

The proposed model is interpretable. The engineers and technicians in the wind industry can trust the model’s accuracy. We have used a confusion matrix to show the classification visually. A classification problem’s prediction outcomes are compiled in a confusion matrix. Count values are used to summarize the number of accurate and inaccurate predictions for each class. Figure 11 shows the confusion matrix obtained after fitting the proposed model. The *x*-axis represents the actual faults, and the y-axis represents the predicted faults. The diagonal of the matrix shows the true predictions.

### 5.2. Receiver Operating Characteristics

**Figure 12:**
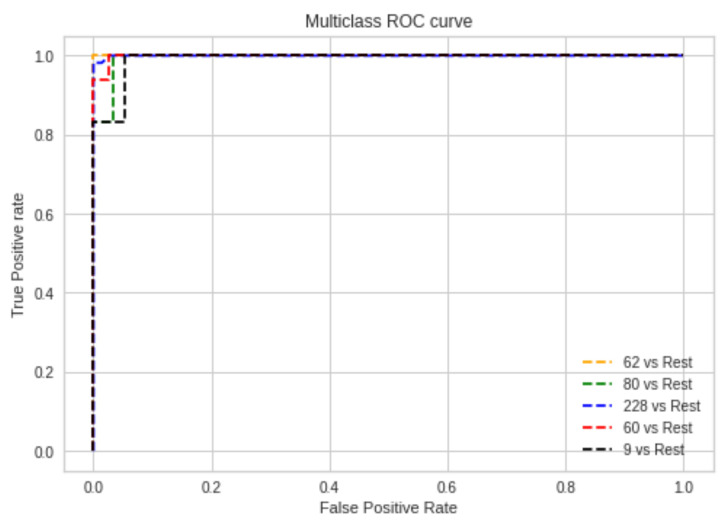
ROC curve for multiclass faults.

For multiclass problems, ROC curves can be plotted using one class versus the rest [43]. To see how well a model performs between sensitivity and specificity, we use a ROC (receiver operating characteristics) plot. Sensitivity is the ability to correctly recognize entries that belong to the positive class. Accurately identifying entries that belong in the negative class is referred to as specificity. Figure 12 shows the ROC curve for multiclass faults, which compares each fault with the rest of the faults.

### 5.3. Precision

Precision is the number of classified correct outputs, or the exactness of the model. It is calculated by using Equation (3), where $T_{p}$ represents true positive values and $F_{p}$ represents false positive values.

(3) $$Precision=\frac{T_{p}}{T_{p}+F_{p}}$$

### 5.4. Recall

Recall is the measure of our model correctly identifying true positives. It is calculated by using Equation (4), where $T_{p}$ represents true positive values and $F_{n}$ represents false negative values.

(4) $$Recall=\frac{T_{p}}{T_{p}+F_{n}}$$

### 5.5. Accuracy

Accuracy is the percentage of correctly predicted outputs. It measures how many positive and negative observations were correctly classified. It is calculated by using Equation (5), where $T_{p}$ represents true positive values, $T_{n}$ represents true negative values, $F_{p}$ represents false positive values, and $F_{n}$ represents false negative values.

(5) $$Accuracy=\frac{T_{p}+T_{n}}{T_{p}+F_{p}+F_{n}+T_{n}}$$

### 5.6. F1 Score

F1 score is the average of precision and recall. It combines precision and recall into one metric by calculating the harmonic mean between those two. It is calculated by using Equation (6), where $T_{p}$ represents true positive values, $F_{p}$ represents false positive values, and $F_{n}$ represents false negative values.

(6) $$F_{1}=\frac{2T_{p}}{2T_{p}+F_{p}+F_{n}}$$

We compared our proposed model with other state-of-the-art machine learning classifiers. We compare the proposed stacking ensemble classifier with AdaBoost, KNN, logistic regression, quadratic discriminant analysis, naive Bayes, and decision tree classifier. Some of the machine learning and deep learning models perform better individually; however, a stacking ensemble learns the most effective way to integrate the predictions from various effective deep learning or machine learning models. We have compared the performance of individual models with the proposed model. Table 2 shows a comparison of accuracy, precision, recall, and F1 score. AdaBoost, KNN, and logistic regression performed best as compared to other models, so we used these three classifiers and ensembled them using the stacking ensemble method. Machine learning models can perform reasonably well on their own, but in this study, we discovered that combining several of the best-performing ML models can produce even better results. The best feature selection, for which we used a confusion matrix, is a further factor in increasing accuracy. The proposed model performs better than the individual models when measured against a variety of evaluation metrics.

## 6. Conclusions

With a focus on renewable energy sources, the reliability and efficiency of wind turbines have become an essential issue in enhancing the total installed capacity of wind turbines. Improved performance requires upgrades in various parts of wind turbines. This paper demonstrates a wind turbine fault detection method based on the AdaBoost, K-nearest neighbors, and logistic regression-based stacking ensemble (AKL-SE) classifier. The anomaly identification model proposed in this paper can identify the fault state of a wind turbine. A new stacking classifier-based ensemble classification method is proposed for fault diagnosis. We have used data from the supervisory control and data acquisition (SCADA) system to improve wind turbine fault detection accuracy. This article provides a solution to identify multi faults in a wind turbine. We used three classification models, AdaBoost, K-nearest neighbors, and logistic regression, as the primary format for generating output. The output of these three classifications was used as the input to the meta-model of the logistic regression classifier. We compared our proposed model with other advanced machine learning classifications. We compared the proposed stack ensemble classifier with AdaBoost, a CNN, logistic regression, quadratic discrimination analysis, naive Bayes, a recurrent neural network, and decision tree classifier. Accuracy, precision, recall, and F1 scores were also compared. Since AdaBoost, KNN, and logistic regression worked better than other models, we combined these three classifications using the stacking ensemble method.

The data used for this study were obtained from a single wind turbine; however, the proposed approach is transferable and generalizable to multiple wind farms. The proposed method is more robust as compared to the traditional approach. However, some improvements need to be made to enhance trustworthiness. In the future, optimization algorithms can be used to enhance accuracy.

## Figures and Tables

**Table 1:** Hyperparameter settings for the base and meta-models.

Sr #	Parameter	AdaBoost	Logistic Regression	KNN
1	base estimators	none	-	-
2	learning rate	1.0	-	-
3	n estimators/jobs	50	none	−1
4	random state	none	none	-
5	leaf size	-	-	30
6	max iter	-	100	-
7	n neighbors	-	-	5
8	c/p	-	1.0	2
9	weights	none	none	uniform
10	penalty	-	12	-

**Table 2:** Comparison of evaluation matrices to other models.

Sr #	Model	Accuracy	Precision	Recall	F1 Score
1	AdaBoost	0.95	0.91	0.95	0.93
2	K-Nearest Neighbors	0.97	0.97	0.96	0.96
3	Logistic Regression	0.96	0.97	0.96	0.96
4	Quadratic Discriminant Analysis	0.88	0.88	0.88	0.87
5	Naive Bayes	0.68	0.77	0.68	0.65
6	Decision Tree Classifier	0.65	0.66	0.65	0.62
7	Recurrent Neural Network	0.72	0.74	0.71	0.72
8	Stacking Classifier	0.98	0.98	0.98	0.97

**Figure A1:**
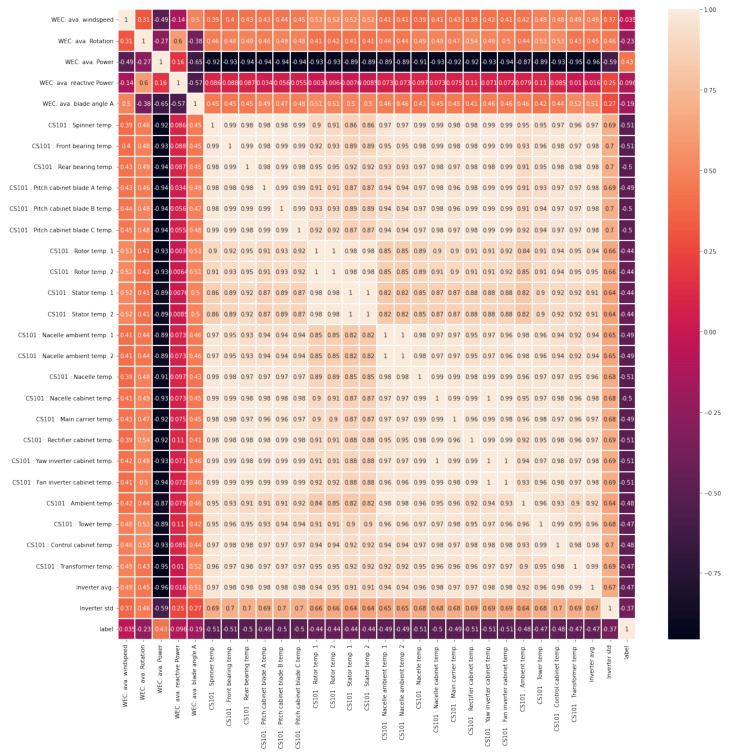
Correlation diagram of features.

## Data Availability

Not applicable.

## Abbreviations

The following abbreviations are used in this manuscript:

SCADA	Supervisory control and data acquisition
O&M	Operation and maintenance
ML	Machine learning
WT	Wind turbines
RES	Renewable energy sources
LR	Logistic regression
MLP	Multi-layer perceptron
SVM	Support-vector machine
LSTM	Long short-term memory
CMS	Condition monitoring system
WEC	Wind energy converter
KNN	K-nearest neighbors
AUC	Area under the curve
ROC	Receiver operating characteristics

## Appendix A

A correlation plot of all the variables is the first step in any factor analysis to evaluate whether some variables are useful or too correlated with others. Figure A1 shows the Correlation diagram of features.
